# Non-invasive urine testing of *EGFR* activating mutation and T790M resistance mutation in non-small cell lung cancer

**DOI:** 10.1186/s40164-016-0052-3

**Published:** 2016-08-08

**Authors:** David Berz, Victoria M. Raymond, Jordan H. Garst, Mark G. Erlander

**Affiliations:** 1 Beverly Hills Cancer Center, 8900 Wilshire Boulevard, Beverly Hills, CA 90211 USA; 2 Trovagene, Inc, 11055 Flintkote Avenue, San Diego, CA 92121 USA

**Keywords:** ctDNA, Circulating tumor DNA, Lung cancer, *EGFR*, Targeted therapy, Liquid biopsy, Cell free DNA, Diagnostics, Urine

## Abstract

**Background:**

The increasing understanding of non-small cell lung cancer (NSCLC) biology over the last two decades has led to the identification of multiple molecular targets. This led to the development of multiple targeted therapies in the primary and secondary resistance setting and the epidermal growth factor receptor (*EGFR*) gene remains the most frequently observed molecular target in NSCLC. Tissue biopsies remain the standard for the identification of such *EGFR* mutations. Obtaining serial tissue biopsies, especially in the secondary resistance setting is associated with multiple medical and logistical challenges. Utilizing circulating tumor DNA (ctDNA) fragments for molecular analysis can overcome these challenges and aid in therapeutic decision-making.

**Case presentation:**

Here we present a present a 72-year-old Korean woman with metastatic, *EGFR* L858R mutated bronchogenic adenocarcinoma. She developed skeletal progression on treatment with first and second generation tyrosine kinase inhibitors (TKIs). Repeated biopsies failed to provide informative molecular test results. A novel urine ctDNA assay was utilized and confirmed T790M positive status. The patient was started on a third generation TKI, which led to a measurable clinical response.

**Conclusions:**

Utilization of urine liquid biopsies for *EGFR* diagnostics are feasible and provided critical clinical information in this patient’s case. Urine liquid biopsy represents a viable alternative to tissue biopsy, particularly in the secondary resistance setting, when tissue is not available for molecular testing.

## Background

Advances in targeted drug development, focused on somatic mutations have significantly changed the therapeutic landscape of lung cancer. In non-small cell lung cancer (NSCLC), constitutively activating *EGFR* (epidermal growth factor receptor) mutations occur in about 11–16 % of patients from the United States and Europe [1–3]. In patients of Asian descent, the mutation frequency is higher, an estimated 61.1 % in females and 44.0 % in males [4]. The identification of patients with activating *EGFR* mutations is clinically meaningful as treatment naïve patients are exquisitely sensitive to small molecule tyrosine kinase inhibitors (TKIs). Initial response rates to first and second generation TKIs are in excess of 50 % [5]. Unfortunately, responses are generally of limited duration with a progression free survival of 10–11 months [6–9]. Several acquired TKI resistance mechanisms have been described, with more than half of the patients developing an *EGFR* exon 20 T790M mutation [10].

Third generation *EGFR* TKIs are uniquely designed for use in patients whose tumors harbor the T790M resistance mutation [11, 12]. Clinical trials have demonstrated excellent response rates to these drugs [13, 14], leading to Food and Drug Administration approval of osimertinib for use in this patient population. However, clinical uptake of these targeted therapeutics is hindered by challenges in obtaining tissue for molecular analysis in the secondary resistance setting. These challenges include lesion inaccessibility, patient performance status, and procedure coordination [15]. Tumor heterogeneity, sample purity, and pre-fixation/fixation artifacts further complicate the interpretation of genomic results [16, 17].

This calls for novel, alternative molecular methods for assessment of the *EGFR* mutation status in the secondary resistance setting.

Here we present a case of a 72-year-old female of Korean descent, who developed clinical resistance to first and second line *EGFR* TKIs. Circulating tumor DNA (ctDNA) isolated from urine was evaluated for the presence of *EGFR* mutations.

## Case presentation

**Fig. 1:**
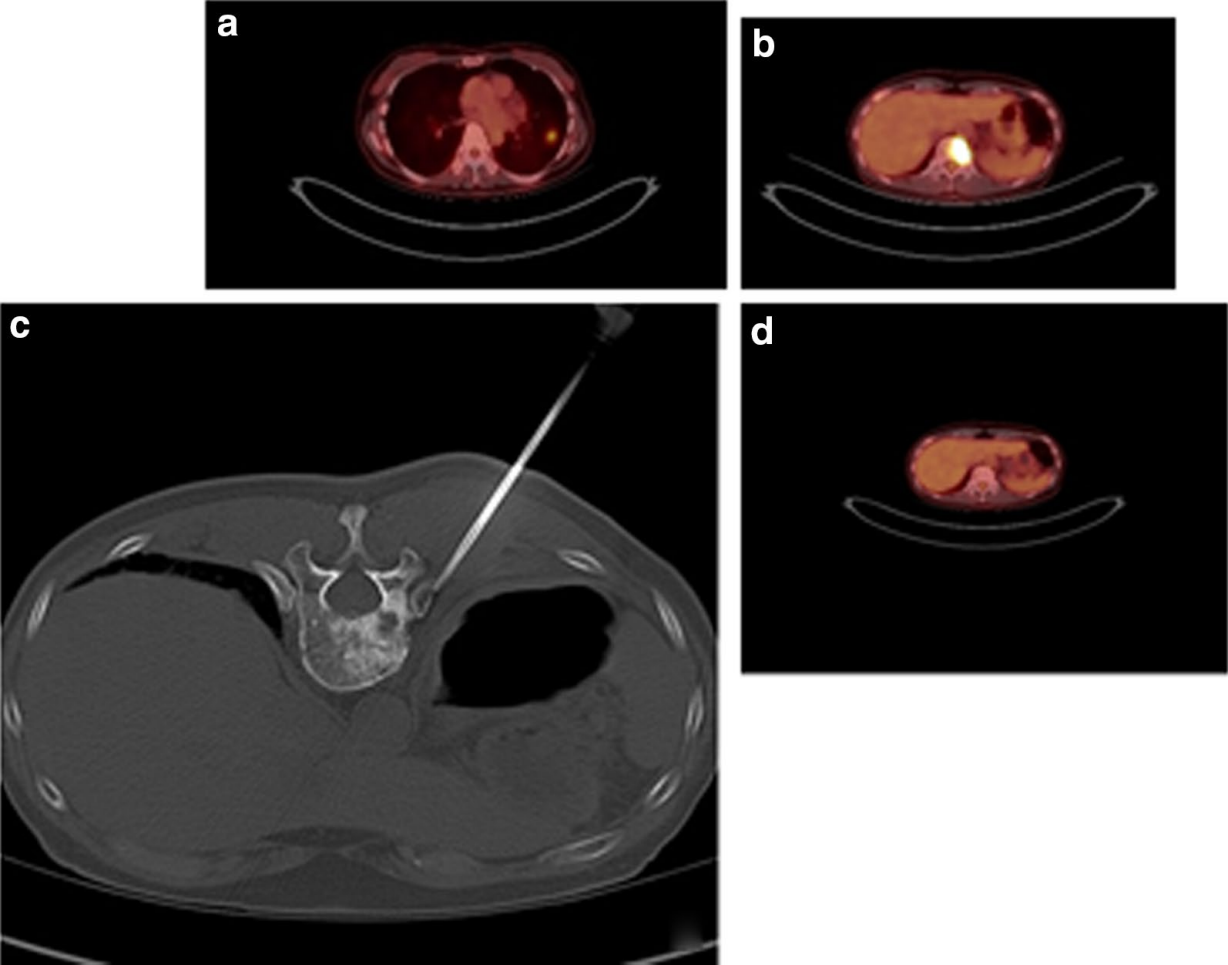
**a** Diagnostic imaging from September 2013, 2 cm left lower lobe lesion, multilevel mediastinal involvement, and numerous hypermetabolic skeletal lesions. **b** May 2014 PET-CT demonstrating new hypermetabolic lesion at T12. **c** January 2015 attempted CT guided biopsy of T12 lesion. **d** August 2015 PET-CT demonstrating response to third generation TKI following identification of *EGFR* resistance mutation

In September 2013, a 72-year-old never smoking female of Korean descent presented with progressive shortness of breath. Chest X-ray and positron emission tomography–computed tomography (PET-CT) were notable for a 2.0 cm dominant left lower lung lobe lesion with multilevel mediastinal disease and widespread involvement of the axial skeleton (Fig. 1a).

CT guided needle biopsy demonstrated a well-differentiated bronchogenic adenocarcinoma (CK7 positive, CK20 negative, TTF-1 positive). Tissue analysis was performed and was positive for the *EGFR* L858R activating mutation in exon 21 while *ALK* was wild type. She was diagnosed with stage IV, T1a, N2, M1b bronchogenic adenocarcinoma.

Given the presence of a somatic *EGFR* activating mutation, the patient started single agent erlotinib as first line therapy, which was well tolerated. Partial response was confirmed in December 2013 when PET-CT revealed a decrease in both size and fludeoxyglucose (FDG) avidity of the dominant left lower lobe lung lesion, as well as the mediastinal and skeletal metastases. Lesions remained stable by PET-CT in March 2014.

A May 2014 PET-CT noted continued decrease in size and metabolic activity of the left lower lung lobe lesion, but a new skeletal lesion was identified at T12 (Fig. 1b). The option of re-biopsy was discussed, specifically to identify emergence of acquired resistance mechanisms for consideration of alternative therapies, but the patient declined and opted to continue with erlotinib therapy. When follow up PET-CT in August 2014 showed further skeletal progression, especially at T12, she agreed to a core needle biopsy of the skeletal lesion (Fig. 1c). The sample was submitted for massively parallel sequencing, but was insufficient for comprehensive genomic evaluation. Given that molecular testing did not reveal a mechanism of somatic resistance, the patient continued on erlotinib therapy.

In September 2014, the patient developed subtle discomfort over her spine without neurologic dysfunction. Treatment discussions included radiation therapy and second-generation TKI therapy. She opted for the latter and treatment with afatinib was initiated. This treatment improved her back pain, but her course was complicated by a hospital admission for grade 4 diarrhea. A PET-CT in November 2014 demonstrated further progression at T12 and a repeat biopsy of the T12 lesion was performed in January 2015. *EGFR* allelotyping was attempted, but results were again inconclusive due to insufficient amount of extracted tumor DNA.

In February 2015, her *EGFR* somatic resistance status remained unknown and she started carboplatin and paclitaxel chemotherapy subsequently transitioning to carboplatin and pemetrexed. Increasing asthenia, nausea and vomiting, and two admissions for neutropenic sepsis within 4 weeks led to discontinuation of cytotoxic therapy.

**Fig. 2:**
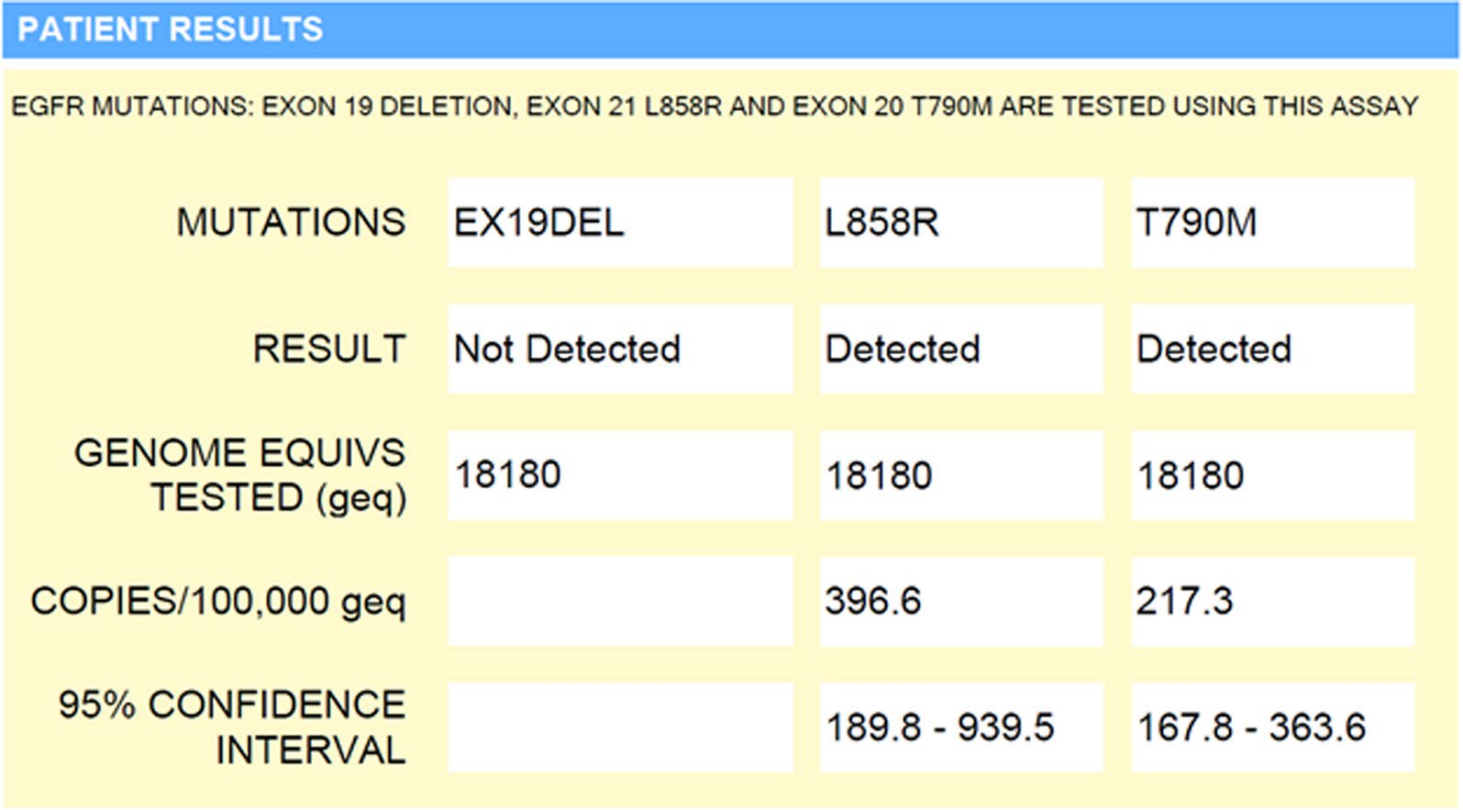
Urine ctDNA test results confirming presence of *EGFR* L858R activating mutation and emergence of *EGFR* T790M resistance mutation

**Fig. 3:**
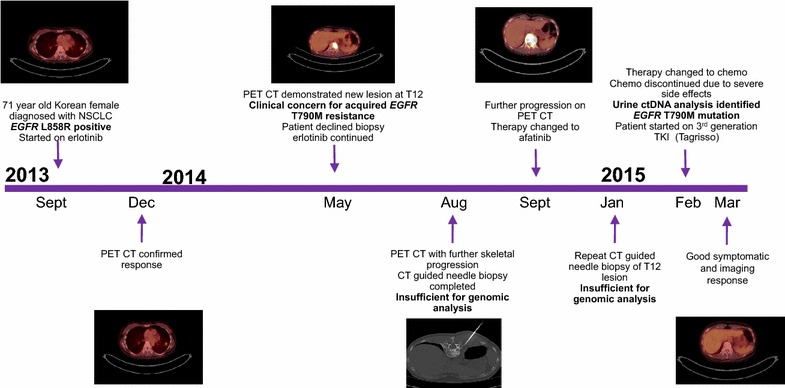
Timeline of therapy, diagnostic evaluation, and patient status

The option of treatment with third generation TKI therapy was discussed, but tissue biopsy was thus far uninformative in determining emergence of the *EGFR* T790M resistance mutation. Liquid biopsy ctDNA analysis was considered as an alternative assessment of resistance. Urine ctDNA analysis was performed and confirmed the L858R activating mutation at 397 copies [per 100,000 genome equivalents (GEq)]. The analysis was also positive for *EGFR* T790M (217 copies per 100,000 GEq) (Fig. 2). With identification of the resistance mutation, nearly 10 months after initial clinical suspicion of acquired resistance, patient became eligible for and initiated on a third generation TKI inhibitor, osimertinib. She showed good symptomatic and radiographic response (Figs. 1d, 3).

## Conclusions

Advances in DNA sequencing technologies have allowed for sophisticated analysis of the tumor genomic landscape [18]. The identification of specific somatic driver mutations influences therapeutic decision making, permitting a personalized approach to oncology care. Tumor tissue has long been considered the best source of material for molecular analysis. However, the serial tissue biopsies needed to obtain the most up to date tumor molecular signatures are often difficult to obtain and can be associated with significant morbidity, thus hindering the potential impact of these targeted therapeutics. A 2014 study found 19.3 % of patients who undergo a lung biopsy experience an adverse event [19]. It is estimated that approximately 25 % of patients who undergo tissue biopsy for genomic evaluation fail to get informative test results due to poor DNA quantity, quality or inadequate tissue sample obtained [20]. Issues such as intra and inter tumor heterogeneity further complicate the interpretation of molecular results from tissue diagnosis. Additionally, in patients in whom tissue and DNA quality and quantity are sufficient, turn-around time for results can be lengthy, often 30 or more days, impacting the potential of these targeted therapies [20].

The presented case highlights a common clinical scenario when multiple tissue biopsies were completed and failed to obtain critical clinical information, delaying the time to clinically impactful information by more than 10 months. Systemic circulating tumor DNA (ctDNA) has recently emerged as a clinically viable alternative to a tissue biopsy [21]. In patients with cancer, plasma ctDNA contains genomic mutations that are concordant with the primary tumor and ctDNA has greater representation of mutation burden than single tissue biopsies [22–26]. Systemic ctDNA fragments are able to negotiate the glomerular filter and can reliably be detected in urine [27]. Recent publications have demonstrated concordance between urine ctDNA and tissue biopsy, and urine ctDNA and plasma ctDNA making urine ctDNA a viable alternative to tissue biopsy and plasma [27–29]. Reckamp et al. [30] published an interim data set of 63 patients with metastatic NSCLC, progressing on first or second line TKI therapy, eligible for a third generation TKI (rociletinib). Tissue, plasma, and urine samples were collected from patients in order to determine *EGFR* T790M status. The sensitivity of urine and plasma versus tissue in detecting T790M was 93 % (13/14) and 93 % (38/41) respectively. Additionally, the liquid biopsy samples provided information on mutational status in patients who had tissue samples inadequate for mutational analysis, and those thought to be negative by tissue analysis. The expanded dataset of 213 patients with matched tissue and urine was presented by Wakelee et al. [31] at the 2016 American Society of Clinical Oncology and demonstrated an 81.1 % sensitivity for urine versus tissue. Furthermore, the response rate to the third generation TKI was similar across all three sample types tested.

Urine ctDNA analysis provides the advantage of being a completely noninvasive sample type, eliminating other clinical and logistical challenges related to acquisition of blood from patients with cancer, such as difficulties with phlebotomy due to fragile, small or difficult to locate veins. Additionally, because urine is a readily available sample type, acquisition does not necessitate the procedure coordination or time in a phlebotomy lab for a tissue biopsy or blood sample respectively. In Reckamp et al. [30] and the expanded dataset presented by Wakelee et al. [31] the urine sample was collected at a time of day based on patient convenience. Kinetic urine studies are in progress to determine the best time of day at which to collect the urine sample.

The urine and plasma assays have a lower limit of detection of one mutant copy per 18,181 GEq for *EGFR* exon 19 deletions and L858R and two copies per 18,181 GEq for T790M [30]. Quantitation of the baseline urine ctDNA mutation burden could enable longitudinal monitoring of mutation load for assessing response to treatment [23, 32–34]. Reckamp et al. [30] published data demonstrating dynamic changes in *EGFR* T790M mutation load within 21 days in patients with metastatic NSCLC starting on a third generation TKI (rociletinib). In a pilot study, Hussain et al. [35] demonstrated that monitoring for early emergence of T790M in patients with *EGFR* positive NSCLC identified the mutation up to 3 months prior to radiographic progression. Ongoing work will demonstrate the ability of ctDNA quantitation to be used to detect resistance mechanisms in advance of imaging and as an early response biomarker.

This case demonstrates the clinical utility of urine ctDNA analysis. Targeted urinary *EGFR* mutation analysis provided a safer and less invasive source of molecular information, eliminating tissue biopsy related patient morbidity. *EGFR* T790M identification was instrumental in the optimal choice of therapy. Utilizing this technology earlier in treatment would have provided critical information in a more timely fashion and could have eliminated the need for multiple uninformative tissue biopsies. Urinary ctDNA analysis allows for a more comprehensive analysis of the tumor mutation burden as compared to tissue biopsy and should be considered in treatment decision-making.

## Abbreviations

*EGFR*: epidermal growth factor
ctDNA: circulating tumor DNA
NSCLC: non-small cell lung cancer
TKI: tyrosine kinase inhibitor
FDG: fludeoxyglucose
PET-CT: positron emission tomography–computed tomography
GEq: genome equivalents
cm: centimeter
